# Identification of EBV infection in adults with egg specific food allergy

**DOI:** 10.1186/1743-422X-10-9

**Published:** 2013-01-04

**Authors:** Yang Pan, Zhiyang Nie, Yuan Zhang, Kuo Zhang, Jinming Li, Lunan Wang

**Affiliations:** 1 National Center for Clinical Laboratories, Beijing Hospital of the Ministry of Health, NO.1 Dahua Road, Beijing, People’s Republic of China; 2 Graduate School, Peking Union Medical College, Chinese Academy of Medical Sciences, Beijing, People’s Republic of China; 3 Department of Transfusion, Beijing Hospital of the Ministry of Health, Beijing, People’s Republic of China

**Keywords:** Epstein-Barr virus, Food allergy, Microrna, Specific IgE

## Abstract

**Background:**

Food allergy has been reported increasingly around the world during the past several decades. Epstein-Barr virus (EBV), a common herpesvirus with high infection rate, is now suspected to be a risk or protective factor in food allergy. The aim of the study was to investigate the possible role of EBV infection in IgE-mediated food allergy.

**Methods:**

34 patients with an egg allergy and 34 healthy controls participated in this study. Egg allergy was confirmed by open-food challenge. Serum anti-viral capsid antigen (VCA), anti-Epstein-Barr nuclear antigen 1 (EBNA-1) IgG and egg specific (yolk and white)-IgE levels were evaluated by enzyme linked immunosorbent assay (ELISA). At the same time, EBV DNA as well as viral miRNAs in these samples was quantified by real-time PCR.

**Results:**

The results showed that serum anti EBNA-1 IgG and two viral miRNAs (miR-BART1-5p and miR-BART7) were highly expressed in patients with egg allergy compared with healthy controls (p < 0.05, < 0.001 and < 0.01, respectively). Moreover, the expressions of anti EBNA-1 specific IgG, miR-BART1-5p and miR-BART7 positively correlated with the level of egg-specific IgE (p < 0.05, < 0.01 and < 0.01, respectively). The differences in anti VCA IgG concentration and EBV DNA copy number between the allergy patients and control individuals were not statistically significant.

**Conclusions:**

The high expression of EBV-specific antibody and miRNAs indicated that EBV infection might play a promoting role in IgE-mediated egg food allergy, and viral miRNAs-related immunomodulatory pathway was likely involved in this allergy process.

## Background

Food allergy (FA) refers to a series of adverse reactions to foods that are immunologically mediated. Over the past several decades, the prevalence of FA has markedly increased in developed countries, and become an important public health problem [1,2]. Because there is no effective treatment to prevent or ease allergy reactions currently, identification of possible risk factors for FA is of great clinical importance.

The occurrence of FA is based on the disorder of a complex immunoregulatory network, including host- or antigen-specific properties, dietary, as well as other environmental factors. The etiology of FA has not been completely elucidated yet, except that children with allergic parents more often get allergy; however, Epstein-Barr Virus (EBV) infection has been implicated in influencing IgE-mediated sensitization in early childhood. Some studies have suggested a food allergy-promoting effect [3], whereas others showed an age dependent allergy-protective role of EBV: only the children who acquired EBV infection before two years of age can benefit from it [4,5]. Further investigations are needed to verify the precise role of EBV infection in food allergic disorders. Moreover, most studies published so far have focused on early EBV infection in infants or children, while the possible relationship between EBV infection and adult food allergy is rarely reported.

Therefore, we propose to conduct an investigation on the relationship between EBV infection and FA. Considering egg protein is one of the most common food allergens around the world [6,7], current study recruited patients with egg specific FA. EBV-specific antibodies, viral DNA and viral microRNAs (miRNAs) are included in this study. EBV viral miRNAs are a set of miRNAs derived from EBV genome. The expression of these miRNAs is often associated with various disease states triggered by viral infections [8,9]. Assessing the role of these miRNAs in the pathophysiology and their possible application as biomarkers for different phases of infection is a focus of current researches. Here we used serological as well as molecular methods to evaluate the association of EBV infection and its possible role in FA.

The aim of this study is to explore the possible correlation between EBV infection and IgE-mediated egg-specific food allergy in Chinese adults.

## Results

### EBV infection in the allergic and controls

**Figure 1:**
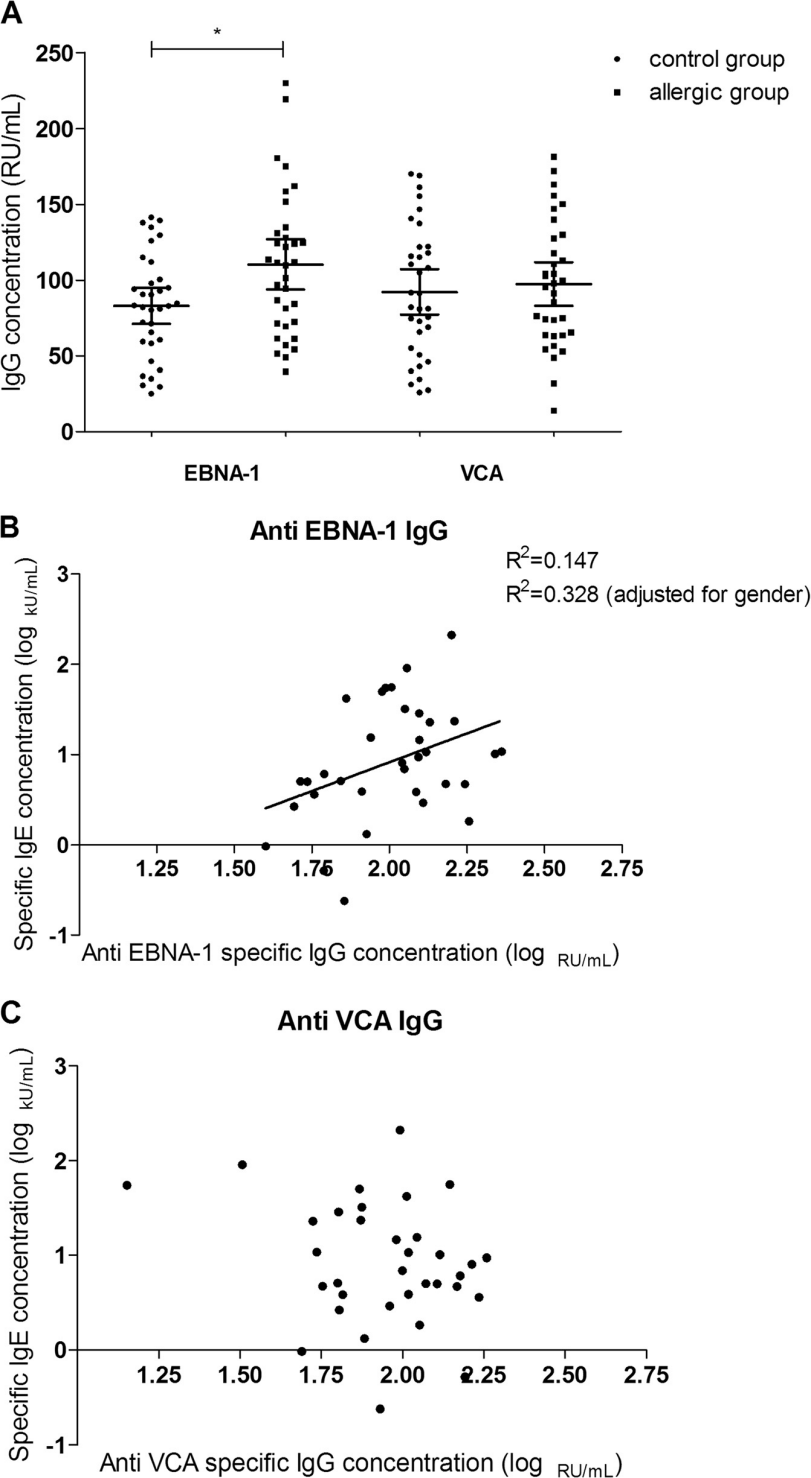
**Anti EBNA-1 and anti VCA IgG concentrations in food allergic and control group. (A)** Median and interquartile range (error bars) of EBV EBNA-1 and VCA IgG concentrations in 34 food allergic patients and 34 healthy controls. * p < 0.05, by t-test; **(B)** the linear regression between egg-specific IgE and anti EBNA-1 IgG (p < 0.05; p < 0.01, (adjusted for gender), by multiple linear regression); and **(C)** the linear regression between egg-specific IgE and anti VCA IgG (p > 0.05). R^2^ values are indicated.

The concentrations of anti-viral capsid antigen (VCA) and anti-Epstein-Barr nuclear antigen 1 (EBNA-1) specific IgG antibodies are summarized as median (interquartile range) (Figure 1A and Table 1 and Additional file 1: Table S1). The mean level of anti EBNA-1 IgG was 83.13 RU/mL (59.10 to 103.38 RU/mL, interquartile range) in controls and 110.96 RU/mL (71.13 to 132.34 RU/mL) in allergic groups. Meanwhile, the concentration of anti VCA IgG was 86.80 RU/mL (53.97 to 122.05 RU/mL) in controls and 96.98 RU/mL (63.90 to 128.41 RU/mL) in allergic groups. Compared with the healthy control group, the allergic group showed a higher level of anti EBNA-1 IgG (p < 0.05). However, such a difference was not observed in anti VCA IgG between the two groups (p = 0.681). Notably, no anti-VCA IgM positive sample was identified in both allergic and healthy control group, indicating the past infection of EBV in all participators.

**Table 1:** Characteristics of allergic and control group

	**Total number (male %)**	**Age (years)**	Egg SPT weal diameter (mm)*	Egg-specific IgE (kU/L)*	Anti EBNA-1 IgG (RU/mL)*	Anti VCA IgG (RU/mL)*	**Anti-VCA IgM**	**miR-BHRF1-1 (−ΔCt)**	**miR-BART1-5p (−ΔCt)**	**miR-BART7 (−ΔCt)**
Controls	34 (41.18)	46.91 ± 9.02	ND	< 0.35	83.13 (59.10-103.38)	86.80 (53.97-122.05)	NG	10.99 ± 1.66	10.78 ± 1.39	10.72 ± 1.43
					25.20-141.32	26.00-170.00				
Egg allergic	34 (41.18)	48.47 ± 8.89	4.0 (3.5-5.5)	7.51 (3.78-24.85)	110.96 (71.13-132.34)	96.98 (63.90-128.41)	NG	11.46 ± 1.77	12.54 ± 1.72	11.95 ± 1.58
			3.0-10.0	0.24-211.46	39.90-230.29	14.23-181.65				
p value	-	0.475^†^	-	-	< 0.05^‡^	0.681^‡^	-	0.266^†^	< 0.001^†^	< 0.01^†^

*, Data expressed as median in top row, interquartile range in the middle, and range in bottom row.

†, by Student t-test; ‡, by Mann–Whitney U-test.

SPT, skin prick test. ND, not determined. NG, negative.

Serum DNA samples from 34 allergic patients and 34 controls were analyzed using commercial real-time quantitative PCR system. Each of the two groups had one EBV DNA positive sample, but the viral loads were relatively low. The positive sample from the allergic group had a DNA concentration of 5.6×10^3^ copies/mL, while that from the control group was 5.6×10^4^ copies/mL. All serum DNA samples (patients and controls) were amplifiable using β-actin PCR, to confirm the quality of the serum-extracted DNA.

Samples with anti EBNA-1 IgG and/or anti VCA IgG seropositivity (≥ 22 RU/mL and ≥ 22 RU/mL, respectively), or with EBV DNA positivity, were considered to have been infected with EBV [10]. Data for the current study showed that all the allergic patients and the healthy controls were infected.

### Serum EBV viral miRNA

**Figure 2:**
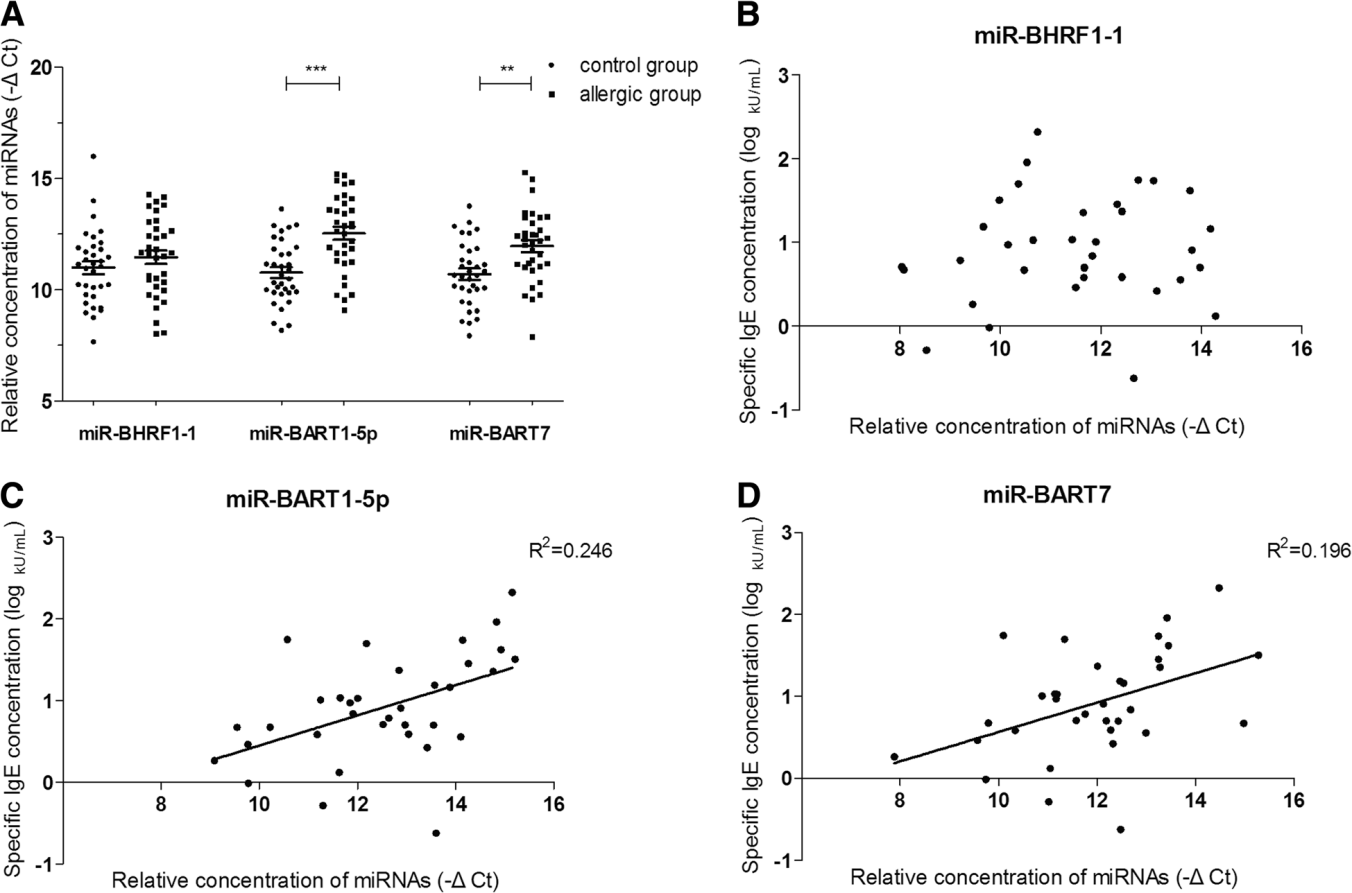
**Viral miRNAs expression in food allergic and control groups. (A)** Mean + (5–95) percentile (error bars) of EBV miR-BHRF1-1, miRBART1-5p and miR-BART7 concentrations in 34 food allergic patients and 34 healthy controls. Viral miRNAs concentration is normalized to that of U6 RNA and is presented as –ΔCt, where Ct is the threshold cycle. **, p < 0.01; ***, p < 0.001, by t test; **(B)** the correlation between egg-specific IgE and miR-BHRF1-1 (p > 0.05, by multiple linear regression); **(C)** between egg-specific IgE and miR-BART1-5p (p < 0.01); and **(D)** between egg-specific IgE and miR-BART7 (p < 0.01). R^2^ values are indicated.

Three EBV coding viral miRNAs, including miR-BHRF1-1, miR-BART1-5p and miR-BART7, were chosen to evaluate the EBV infection in this study. After PCR amplification, melting curve analysis was done to ensure the specificity of test. It showed that miR-BART1-5p had a higher level in the allergic group compared with the control group (p < 0.001, 95% CI 1.004-2.517). The expression of miR-BART7 was similar to that of miR-BART1-5p, increasing significantly in allergic group (p < 0.01, 95% CI 0.520-1.979). In this study, the mean concentration of three viral miRNAs was expressed as a -fold change compared with the control group. Therefore the concentration of miR-BART1-5p and miR-BART7 increased 3.39 and 2.35-fold in allergic patients compared with healthy individuals, respectively. Whereas the miR-BHRF1-1 level only had a mild change between these two groups (1.39-fold increase) and no significant difference was observed (p = 0.266) (Figure 2A and Table 1).

### Correlation analysis between EBV infection and egg-specific IgE

Among 34 patients participated this study, linear regression analysis showed that the anti EBNA-1 specific IgG level was correlated with the egg-specific IgE concentration (p < 0.05, 95% CI 0.168-2.391, R^2^ = 0.147; p < 0.01, 95% CI 0.715-2.843, R^2^ = 0.328 (adjusted for gender)) (Figure 1B and Table 2). Moreover, the positive correlationships between EBV specific miR-BART1-5p or miR-BART7 expression and the level of egg-specific IgE were also proved in current study (p < 0.01, 95% CI 0.068-0.302, R^2^ = 0.246 and p < 0.01, 95% CI 0.049-0.311, R^2^ = 0.196, respectively) (Figure 2C, D and Table 2). However, other EBV infection markers, including anti VCA IgG and viral miR-BHRF1-1 showed no significant up or down tendency in allergic patients with different specific IgE levels.

**Table 2:** The multiple linear regression analysis in allergic patients

	**Slope 95% CI**	**Standardized coefficients**	**R**^**2**^	**p value**
Egg-specific IgE vs. anti EBNA-1 IgG	0.168-2.391	0.383	0.147	< 0.05
	0.715-2.843*	0.532*	0.328*	< 0.01*
Egg-specific IgE vs. miR-BART1-5p	0.068-0.302	0.496	0.246	< 0.01
Egg-specific IgE vs. miR-BART7	0.049-0.311	0.443	0.196	< 0.01

*, adjusted for gender.

CI, confidence interval.

## Discussion

EBV is a large DNA virus of γ herpes subfamily. It infects around 98% of the world’s population [11]. It is well known that EBV exerts a wide range of immune modulating effects, inhibition of apoptosis, and disturbance of the anti-EBV effects of interferon-γ (IFN-γ) within B cells. Furthermore, it also plays an important role in the production of pro-inflammatory cytokines such as tumor necrosis factor-α (TNF-α), interleukin-1 (IL-1) and IL-6 [12]. However, the pathogenic influence of EBV in allergic disorders, such as FA, remains unclear. T_H_1/T_H_2 immune response may play a central role in EBV related food allergy reaction. T_H_1 cytokines induce major histocompatibility complex molecules and activate T cells. Alternatively, T_H_2 cytokines, such as IL-4, IL-6 and IL-10, activate B cells and stimulate antibody production. After the EBV infection, T_H_2 cells can be induced to express IL-10 and its analogue, which stimulate B-cell proliferation and polyclonal antibody production [13,14]. And indeed, an increased level of anti EBNA-1 IgG in egg-specific food allergic patient was proved in current study. Because EBNA-1 is the only viral protein expressed during the first latency phase and VCA is a major capsid antigen only involved in lytic cycle [15], our result shows a higher serological reactivity against EBNA-1 in FA patients, therefore indicates that the reactivation of latent EBV infection may be a promoting factor in allergy reaction. It is noteworthy that Nilsson C et al. and Saghafian-Hedengren S et al. indicated that the acquisition of EBV infection in the first two years of life may reduce the risk of food allergen-specific IgE sensitization in childhood [5,6]. The discrepancy may due to the different age and race of participators, early or past latent infection, the diversity in the medical history, and the accuracy of detection methods. Considering that the study population and EBV infection status are two of the most significant differences between ours and the former studies, it will be interesting to know whether EBV plays a dual role in patients with FA: protecting the infants while harming the adults.

Another important finding in this study is the increased concentration of EBV viral miR-BART1-5p and miR-BART7 in patients with egg FA, which indicated that there might be an association between EBV and FA on molecular level. EBV has been shown to encode at least 40 miRNAs, located in two regions of EBV genome [16]: the BHRF1 miRNAs locate immediately upstream and downstream of the BHRF1 open reading frame, while the BART miRNAs lie within the intronic regions of the BARTs. In this study, we observed a significant increase of miR-BART1-5p and miR-BART7 levels in patients with FA. Previous study has reported that the expression of the BART miRNA cluster was a characteristic of latent EBV infection and this expression was relatively constant during virus replication [17]. In contrast, miR-BHRF1-1, which showed constant expression between patients and controls in this study, is strongly induced in lytic cycle and depended on viral DNA replication [18]. Thus, we hypothesized that egg food allergy was most likely to occur in EBV latent infection, and miR-BART1-5p and miR-BART7 may have particular influence in allergy individuals.

It has been established that viral miRNAs could negatively regulate the expression of both human and viral genes through post-transcriptional mechanisms. Although most targets of EBV viral miRNAs are still not clear, some of them have been identified previously. For example, viral latent membrane protein 1 (LMP1) is the target of miR-BART1-5p, miR-BART16-5p and miR-BART17-5p [19]. Several studies suggest that miR-BART1-5p negatively regulates LMP1 gene expression through binding to specific sequences in 3’ UTR [20]. Meanwhile, LMP1 shows multiple roles in immune regulation. It acts as a constitutively active receptor that mimics activated CD40, regulates different signaling pathways including NF-κB, AP-1, p38-MAPK/ATF and STAT, interacts with multiple cytokines such as IL-10, and plays a critical role in EBV-induced B-cell transformation [21-24]. It is reasonable to speculate that EBV latent miRNAs, such as miR-BART1-5p, may influent immune regulation by multiple ways and finally trigger or aggravate the food allergy reaction in adults. To verify this hypothesis and to further reveal the roles of the EBV miRNAs in FA, considerable investigations still need in this area.

In order to obtain more extensive information about the relationship between EBV infection and FA, we also analyzed the concentration of EBV DNA in patients and controls. It was noteworthy that, EBV DNA was only detected in very few allergic or healthy samples (one case in each group) and the DNA burdens of these positive samples were relatively low (between 10^3^-10^4^ copies/mL). One potential explanation may be the latent stage of viral infection in patients with FA. Compared with lysis stage, the serum virus load of latency may be 10^4^-10^5^ folds lower, resulting in a negative PCR result (the sensitivity of real-time PCR kit which was used in our study is 10^3^ copies/mL) [25]. This low detected ratio was also observed in previous studies [11].

Serum allergen specific IgE may be the most widely used marker in the diagnosis of FA nowadays. Although its diagnosis and prognostic application is controversial due to the variability of sample source, the lack of standardization, and the potential inconsistence with clinical symptoms [26], the detection of specific IgE is easy to achieve a quantitative evaluation and the positive results against culprit food enables sensitization to be established. Therefore we quantified the egg-specific IgE level by ELISA and correlated this data with both EBV specific antibody titer and the expression of viral miRNAs. Notably, every one of the three EBV markers (anti EBNA-1 IgG, miR-BART1-5p and miR-BART7), which showed a differential expression between allergic patients and healthy control in this study, have a fair correlation with egg-specific IgE. On one hand, these correlations confirm the association between EBV miRNAs and FA; On the other hand, it indicates that many factors, such as EBV viral latent miRNAs, may influence the production of food-specific IgE, other than the allergenicity of culprit food. Some signaling pathways, such as NF-κB and AP-1, may be involved in this IgE producing process, as is discussed above.

Several limitations of this study include the limited number of samples, not a long-term observation, and the exclusion of other kinds of FA patients beside egg protein. Because the prevalence of FA in adults is relatively low, it is difficult to conduct a wider and more extensive study. However, the mechanisms of different food allergen-specific allergy are similar [27], so our finding should also be observed in other food allergen-specific reaction.

In conclusion, we report that patients with egg FA have a relative higher concentration of anti-EBNA IgG, EBV miR-BART1-5p and miR-BART7. Furthermore, the expression level of these infection markers correlates with the concentration of egg-specific IgE. Although the exact molecular mechanisms are yet to elucidate, this is the first study suggests the possible role of EBV infection in promoting the IgE-mediated food allergy reaction in adults. What is more, deeper investigations will be required to determine the potential application of using viral miR-BART1-5p and miR-BART7 in evaluating EBV infection and their role in the pathogenesis of FA.

## Methods

### Study samples

Current study was carried out with the approval of the ethical committee at Beijing Hospital. The study group consisted of 34 out-patients (14 male, 20 female) who had egg-specific FA diagnosed between September 2010 and December 2011 in the Beijing area, and oral informed consent was obtained from each participant. Inclusion criteria were (a) a clinical diagnosis of egg-specific FA; (b) a positive result of EBV infection (EBV seropositivity and/or EBV DNA positivity). The diagnosis of egg-specific FA was based on (a) a recent convincing history of an immediate type clinical reaction to egg with typical symptoms [8]; (b) a positive egg specific (yolk and white) skin prick tests (SPTs) result judged by the 95% predictive value for SPT weal diameter ≥ 3 mm (GREER, USA) [28]; and (c) a positive result of open-food challenge test participate the trail. Open-food challenge test was performed to confirm the diagnosis of egg protein-specific food allergy [29]. Briefly, incremental doses of cooked egg (raw HE yolk and white, simultaneously) were given at 30 mins intervals to get a cumulative dose of 12g. The challenge was stopped when either cumulative dose was tolerated or an objective reaction [development of two or more of erythema, urticaria (distant to the mouth) or angiooedema, rhinoconjunctivitis, wheeze, abdominal pain or vomiting] occurred. Food challenge was performed in hospital by a trained physician and the subjects were observed for at least 2 h after the last dose before being sent home. Exclusion criteria were allergic reaction to other food allergens and no significant response to eggs eliminating treatment.

34 healthy individuals attending for health examination between September 2010 and September 2011 were invited to participate as healthy controls. Questionnaires reported no history of allergy-related illness and that enrolled control individuals were not actively avoiding certain foods (including but not limiting to egg). No positive result of egg-specific IgE (> 0.35 kU/L) was identified in control group. All the enrolled control individuals were EBV infected (EBV seropositivity and/or EBV DNA positivity). The mean age was comparable in allergic and controls group (p = 0.475, by t-test). The female to male ratio was equal in both groups. There was no relationship between age and sex in allergic patients, healthy controls and all the individuals enrolled (p = 0.158, 0.631 and 0.608, by Spearman correlation, respectively).

Serum samples of these participators were prepared after centrifugation and stored frozen at −80°C within one hour of collection. Informed consent was obtained from each subject.

### Egg-specific IgE ELISA

The egg-specific IgE levels are measured by food allergens specific REAST commercial kits (FOOKE, Germany). All tests were completed following the manufacturer’s instructions and each sample was analyzed in duplicate. Egg-specific IgE concentration was determined by a standard curve which was established by a series of calibrators. Specific IgE level over 0.35 kU/L were considered positive [26].

### EBV-specific antibodies ELISA

Anti VCA, Anti EBNA-1 IgG and Anti VCA IgM were assessed by commercially available ELISA kits (Euroimmun, Germany) following the manufacturer’s instructions. For each assay, a standard curve was established by calibrators supplied with the kits, and then used to calculate specific antibody concentration.

### EBV DNA real-time PCR

EBV DNA was extracted from serum samples using the QIAamp DNA Mini Kit (Qiagen, Germany) following the instruction of the vendor. A fragment of β-actin (304 bp) was amplified to confirm successful extraction of DNA and no PCR inhibitors were present. The EBV DNA was quantified by EBV PCR fluorescence quantitative diagnostic kit (Daangene, China) using 7500 Real-Time PCR System (Applied Biosystems, USA). Each sample was analyzed in duplicate to obtain a final measurement expressed as DNA copies per millilitre (copies/mL).

### RNA Isolation

Before RNA extraction, patients’ serum were centrifuged at 16,000 g for 10 min. Total RNA (including small RNA molecules) was extracted from 500 μL serum supernatants by TRIzol reagent (Invitrogen, USA) and mirVana miRNA Isolation Kit (Applied Biosystems, USA) following the instructions. The concentration and purity of the RNA product was measured with ultraviolet spectrophotometer (Eppendorf, Germany).

### Real-time RT-PCR

EBV coding miRNA (EBV-miR-BHRF1-1, EBV-miR-BART1-5p and EBV-miR-BART7) sequences were obtained from the Sanger miRNA Registry (http://microrna.sanger.ac.uk) and stem-loop RT primers were designed based on these sequences as previously described [30]. The miRNA expression levels were quantified by real-time PCR using PrimeScript RT reagent Kit (Takara, Japan) and SYBR Premix Ex TaqII Kit (Takara, Japan). Briefly, approximate 50ng of small RNA from each sample was reverse-transcribed to cDNA with stem-loop RT primer and U6 Reverse primer. Subsequently, real-time PCR was performed on StepOnePlus Real-Time PCR System (Applied Biosystems, USA). All reactions were run in triplicate. Ct data were determined by default threshold settings. In this study, U6 RNA was chosen as a miRNA internal control. The relative expression levels of miRNAs were calculated in 2^–ΔCt^ method [31], and the differences of miRNA concentration between allergy group and control group were expressed as -fold changes.

### Statistical analysis

Values were expressed as the mean ± SD. Differences between two groups were analyzed by the Student t-test. Antibody (egg-specific IgE, anti EBNA-1 IgG and anti-VCA-IgG) levels had skewed distributions, so a median (interquartile range) and Mann–Whitney U-test, were applied to analyze the difference between groups. Correlation was assessed by multiple linear regression (adjusted for gender and age). The data with skewed distributions (egg-specific IgE, anti EBNA-1 IgG and anti-VCA-IgG) were converted to logarithmic form for linear regression. All statistical analyses were performed using SPSS 17.0 statistics program (SPSS Inc, USA). A p value <0.05 (two tailed) was considered statistically significant.

## Competing interests

This study was supported by the National High Technology Research and Development Program of China (863 program No.2011AA02A116).

## Authors’ contributions

YP conducted the experiments, performed the statistical analysis and drafted the manuscript. ZN participated in the design of the study, collected specimens and data and modified the manuscript. YZ & KZ completed the assay for the specimens. JL & LW generated the concept for the study, participated in its design and coordination. All authors read and approved the final manuscript.

## Supplementary Material

Additional file 1 **Table S1.** The original data of FA patients. Click here for file

## References

[B1] Mullins RJ Paediatric food allergy trends in a community-based specialist allergy practice, 1995–2006 Med J Aust 2007 186 618 621 17576175 10.5694/j.1326-5377.2007.tb01077.x

[B2] Gupta R Sheikh A Strachan DP Anderson HR Time trends in allergic disorders in the UK Thorax 2007 62 91 96 10.1136/thx.2004.038844 16950836 PMC2111268

[B3] Okudaira H Mori A Concepts of the pathogenesis of allergic disease: possible roles of Epstein-Barr virus infection and interleukin-2 production Int Arch Allergy Immunol 1999 120 177 184 10.1159/000024265 10592462

[B4] Nilsson C Linde A Montgomery SM Gustafsson L Nasman P Blomberg MT Lilja G Does early EBV infection protect against IgE sensitization? J Allergy Clin Immunol 2005 116 438 444 10.1016/j.jaci.2005.04.027 16083803 PMC7119037

[B5] Saghafian-Hedengren S Sverremark-Ekstrom E Linde A Lilja G Nilsson C Early-life EBV infection protects against persistent IgE sensitization J Allergy Clin Immunol 2010 125 433 438 10.1016/j.jaci.2009.09.033 19963258

[B6] Eggesbo M Botten G Halvorsen R Magnus P The prevalence of allergy to egg: a population-based study in young children Allergy 2001 56 403 411 10.1034/j.1398-9995.2001.056005403.x 11350303

[B7] Boyce JA Assa'ad A Burks AW Jones SM Sampson HA Wood RA Plaut M Cooper SF Fenton MJ Arshad SH Guidelines for the Diagnosis and Management of Food Allergy in the United States: Summary of the NIAID-Sponsored Expert Panel Report J Allergy Clin Immunol 2010 126 1105 1118 10.1016/j.jaci.2010.10.008 21134568 PMC4241958

[B8] Gourzones C Gelin A Bombik I Klibi J Verillaud B Guigay J Lang P Temam S Schneider V Amiel C Extra-cellular release and blood diffusion of BART viral micro-RNAs produced by EBV-infected nasopharyngeal carcinoma cells Virol J 2010 7 271 10.1186/1743-422X-7-271 20950422 PMC2974674

[B9] Imig J Motsch N Zhu JY Barth S Okoniewski M Reineke T Tinguely M Faggioni A Trivedi P Meister G microRNA profiling in Epstein-Barr virus-associated B-cell lymphoma Nucleic Acids Res 2011 39 1880 1893 10.1093/nar/gkq1043 21062812 PMC3061055

[B10] Gulley ML Tang W Using Epstein-Barr viral load assays to diagnose, monitor, and prevent posttransplant lymphoproliferative disorder Clin Microbiol Rev 2010 23 350 366 10.1128/CMR.00006-09 20375356 PMC2863367

[B11] Cohen JI Epstein-Barr virus infection N Engl J Med 2000 343 481 492 10.1056/NEJM200008173430707 10944566

[B12] Kanegane H Wakiguchi H Kanegane C Kurashige T Tosato G Viral interleukin-10 in chronic active Epstein-Barr virus infection J Infect Dis 1997 176 254 257 10.1086/517260 9207376

[B13] Ohnishi E Iwata T Inouye S Kurata T Sairenji T Interleukin-4 production in Epstein-Barr virus-transformed B cell lines from peripheral mononuclear cells of patients with atopic dermatitis J Interferon Cytokine Res 1997 17 597 602 10.1089/jir.1997.17.597 9355960

[B14] Klein SC Kube D Abts H Diehl V Tesch H Promotion of IL8, IL10, TNF alpha and TNF beta production by EBV infection Leuk Res 1996 20 633 636 10.1016/0145-2126(96)00029-X 8913315

[B15] Steven NM Epstein-Barr virus latent infection in vivo Rev Med Virol 1997 7 97 106 10.1002/(SICI)1099-1654(199707)7:2<97::AID-RMV190>3.0.CO;2-M 10398475

[B16] Cai X Schafer A Lu S Bilello JP Desrosiers RC Edwards R Raab-Traub N Cullen BR Epstein-Barr virus microRNAs are evolutionarily conserved and differentially expressed PLoS Pathog 2006 2 23 10.1371/journal.ppat.0020023 PMC1409806 16557291

[B17] Pratt ZL Kuzembayeva M Sengupta S Sugden B The microRNAs of Epstein-Barr Virus are expressed at dramatically differing levels among cell lines Virology 2009 386 387 397 10.1016/j.virol.2009.01.006 19217135 PMC2763627

[B18] Amoroso R Fitzsimmons L Thomas WA Kelly GL Rowe M Bell AI Quantitative studies of Epstein-Barr virus-encoded microRNAs provide novel insights into their regulation J Virol 2011 85 996 1010 10.1128/JVI.01528-10 21068248 PMC3020024

[B19] Choy EY Siu KL Kok KH Lung RW Tsang CM To KF Kwong DL Tsao SW Jin DY An Epstein-Barr virus-encoded microRNA targets PUMA to promote host cell survival J Exp Med 2008 205 2551 2560 10.1084/jem.20072581 18838543 PMC2571930

[B20] Motsch N Pfuhl T Mrazek J Barth S Grasser FA Epstein-Barr virus-encoded latent membrane protein 1 (LMP1) induces the expression of the cellular microRNA miR-146a RNA Biol 2007 4 131 137 10.4161/rna.4.3.5206 18347435

[B21] Ahsan N Kanda T Nagashima K Takada K Epstein-Barr virus transforming protein LMP1 plays a critical role in virus production J Virol 2005 79 4415 4424 10.1128/JVI.79.7.4415-4424.2005 15767441 PMC1061545

[B22] Sloan DD Jerome KR Herpes simplex virus remodels T-cell receptor signaling, resulting in p38-dependent selective synthesis of interleukin-10 J Virol 2007 81 12504 12514 10.1128/JVI.01111-07 17804501 PMC2169026

[B23] Gires O Zimber-Strobl U Gonnella R Ueffing M Marschall G Zeidler R Pich D Hammerschmidt W Latent membrane protein 1 of Epstein-Barr virus mimics a constitutively active receptor molecule EMBO J 1997 16 6131 6140 10.1093/emboj/16.20.6131 9359753 PMC1326297

[B24] Martin J Sugden B The latent membrane protein oncoprotein resembles growth factor receptors in the properties of its turnover Cell Growth Differ 1991 2 600 653 1667088

[B25] Kozic S Vince A Bes JI Rode OD Lepej SZ Poljak M Bozic M Kessler HH Evaluation of a commercial real-time PCR assay for quantitation of Epstein-Barr virus DNA in different groups of patients J Virol Methods 2006 135 263 268 10.1016/j.jviromet.2006.03.013 16650904

[B26] Garcia BE Gamboa PM Asturias JA Lopez-Hoyos M Sanz ML Caballero MT Garcia JM Labrador M Lahoz C Longo AN Guidelines on the clinical usefulness of determination of specific immunoglobulin E to foods J Investig Allergol Clin Immunol 2009 19 423 432 20128415

[B27] Vickery BP Scurlock AM Jones SM Burks AW Mechanisms of immune tolerance relevant to food allergy J Allergy Clin Immunol 2011 127 576 584 10.1016/j.jaci.2010.12.1116 21277624 PMC3233381

[B28] Lieberman JA Sicherer SH The diagnosis of food allergy Am J Rhinol Allergy 2010 24 439 443 10.2500/ajra.2010.24.3515 21144222

[B29] Bindslev-Jensen C Ballmer-Weber BK Bengtsson U Blanco C Ebner C Hourihane J Knulst AC Moneret-Vautrin DA Nekam K Niggemann B Standardization of food challenges in patients with immediate reactions to foods–position paper from the European Academy of Allergology and Clinical Immunology Allergy 2004 59 690 697 10.1111/j.1398-9995.2004.00466.x 15180754

[B30] Schmittgena TD Eun Joo Leea JJAS, Terry S 2007 Real-time PCR quantification of precursor and mature microRNA. Methods, Eltona BACC 10.1016/j.ymeth.2007.09.006 PMC2663046 18158130

[B31] Livak KJ Schmittgen TD Analysis of relative gene expression data using real-time quantitative PCR and the 2(−Delta Delta C(T)) Method Methods 2001 25 402 408 10.1006/meth.2001.1262 11846609

