# Supplementary material for: Identification of EBV infection in adults with egg specific food allergy
Source: Virol J. 2013 Jan 4;10:9. doi: 10.1186/1743-422X-10-9 (PMC3551721; doi:10.1186/1743-422X-10-9)
Supplement: Additional file 1 — Table S1. The original data of FA patients. [file 1743-422X-10-9-S1.pdf]

Supplementary Table. 1

The original data of FA patients.

Sample No.	Gender	Age (years)	Egg SPT weal diameter (mm)	Symptoms in OFC	Egg-specific IgE (kU/L)	Anti-EBNA-1 IgG (RU/mL)	Anti-VCA IgG (RU/mL)	miR-BHRF1-1 (-ΔCt)	miR-BART1-5p (-ΔCt)	miR-BART7 (-ΔCt)
1	male	48	6	E, U	1.84	180.72	112.99	9.45	9.09	7.88
2	male	49	4	Ap, V	4.70	175.44	147.22	10.47	9.55	14.98
3	male	66	4	Ap, V	2.91	128.35	91.33	11.5	9.77	9.57
4	male	44	6	A, Ap	0.97	39.90	49.00	9.78	9.78	9.74
5	male	46	5.5	Ap, V	4.73	152.06	56.82	8.08	10.22	9.79
6	male	49	3.5	U, V	55.87	101.67	140.00	12.74	10.57	10.10
7	male	48	5	U, V	3.84	122.08	65.49	11.66	11.18	10.34
8	male	33	5.5	Ap, E	10.20	219.47	130.27	11.9	11.25	10.88
9	male	55	4	E, W	0.52	61.44	155.89	8.53	11.30	11.02
10	male	55	4	Ap, V	1.32	84.49	76.41	14.28	11.63	11.05
11	male	52	4.5	E, U	10.80	230.29	54.47	11.43	11.65	11.14
12	male	47	4.5	Ap, U	9.41	124.22	181.65	10.15	11.85	11.17
13	male	60	4	Ap, U	10.70	131.41	104.43	10.65	12.01	11.19
14	male	48	6	Ap, V	50.2	94.73	73.85	10.36	12.18	11.34
15	female	46	3	Ap, U	5.11	69.70	63.23	8.04	12.52	11.58
16	female	67	4.5	Ap, V	6.10	61.61	150.32	9.20	12.64	11.76
17	female	43	3.5	E, V	23.6	162.22	74.38	12.42	12.85	12.01
18	female	45	4	E, W	8.11	110.01	163.35	13.81	12.88	12.13
19	female	38	9	Ap, W	5.06	51.61	118.07	13.97	12.97	12.19

20	female	55	10	Ap, E, V	3.91	81.55	104.43	12.42	13.04	12.28
21	female	46	6.5	Ap, V, U	2.66	49.30	63.98	13.11	13.42	12.32
22	female	39	6.5	Ap, U	5.02	54.39	127.79	11.67	13.55	12.43
23	female	35	5	U, V	15.50	86.97	110.84	9.66	13.57	12.47
24	female	53	4.5	Ap, E, U	0.24	71.60	85.45	12.65	13.60	12.48
25	female	44	3.5	U, V	14.60	124.97	95.77	14.18	13.88	12.54
26	female	55	3.5	Ap, E	6.92	111.91	99.91	11.83	11.91	12.68
27	female	45	4	A, V	3.61	57.23	172.01	13.58	14.10	12.99
28	female	48	4.5	Ap, V	54.92	97.21	14.23	13.05	14.14	13.25
29	female	43	3.5	Ap, V	28.61	124.80	63.68	12.32	14.26	13.25
30	female	41	4	Ap, U	22.93	135.13	53.06	11.65	14.76	13.28
31	female	48	4	U, V	91.10	113.98	32.19	10.53	14.83	13.42
32	female	62	3.5	Ap, U	42.13	72.60	102.99	13.77	14.92	13.45
33	female	65	3	Ap, V	211.46	158.75	98.18	10.74	15.15	14.48
34	female	30	3	Ap, U	32.12	112.21	75.05	9.98	15.21	15.28

SPT, skin prick test; OFC, oral food challenge. A, angiooedema; Ap, abdominal pain; E, erythema; R, rhinoconjunctivitis; U, urticaria (distant to the mouth); V, vomiting; W, wheeze.
